# Effects of surface functionalization on the adsorption of human serum albumin onto nanoparticles – a fluorescence correlation spectroscopy study

**DOI:** 10.3762/bjnano.5.212

**Published:** 2014-11-07

**Authors:** Pauline Maffre, Stefan Brandholt, Karin Nienhaus, Li Shang, Wolfgang J Parak, G Ulrich Nienhaus

**Affiliations:** 1 Institute of Applied Physics and Center for Functional Nanostructures, Karlsruhe Institute of Technology (KIT), Wolfgang-Gaede-Strasse 1, 76131 Karlsruhe, Germany; 2 Department of Physics, Philipps University Marburg, Renthof 7, 35037 Marburg, Germany,; 3 Department of Physics, University of Illinois at Urbana-Champaign, 1110 West Green Street, Urbana, IL 61801, USA

**Keywords:** fluorescence correlation spectroscopy, human serum albumin, nanoparticles, protein corona, quantum dots

## Abstract

By using fluorescence correlation spectroscopy (FCS), we have studied the adsorption of human serum albumin (HSA) onto Fe–Pt nanoparticles (NPs, 6 nm radius), CdSe/ZnS quantum dots (QDs, 5 nm radius) and Au and Ag nanoclusters (1–4 nm radius), which are enshrouded by various water-solubilizing surface layers exposing different chemical functional groups (carboxyl, amino and both), thereby endowing the NPs with different surface charges. We have also measured the effects of modified surface functionalizations on the protein via succinylation and amination. A step-wise increase in hydrodynamic radius with protein concentration was always observed, revealing formation of protein monolayers coating the NPs, independent of their surface charge. The differences in the thickness of the protein corona were rationalized in terms of the different orientations in which HSA adsorbs onto the NPs. The midpoints of the binding transition, which quantifies the affinity of HSA toward the NP, were observed to differ by almost four orders of magnitude. These variations can be understood in terms of specific Coulombic interactions between the proteins and the NP surfaces.

## Introduction

In recent years, both scientific and commercial applications of nanoparticles (NPs) and other nanomaterials have been increasing at a rapid pace [1–2]. Human health can be adversely affected by NP exposure. A profound assessment of these risks, however, is not yet available because fundamental interactions of nanomaterials with biomatter are still not fully understood [3–5]. Specifically, further detailed mechanistic knowledge at the molecular level is required.

Due to their small size of 1–100 nm, NPs may spontaneously enter the human body through the lung, gut or skin [6–10]. Upon incorporation, NPs come into contact with extracellular fluids such as blood plasma or lung epithelial lining fluid, which contain a huge variety of dissolved biomolecules including lipids and proteins. These can adsorb onto the NP surface and completely enshroud the NP, forming the so-called “protein corona” [11–15]. Protein adsorption onto NPs is governed by chemical kinetics. Thus, the initial binding events involve predominantly the more abundant and mobile proteins. If these proteins do not bind too tightly (so that their residence times on the NP do not exceed the relevant time scales), they may be subsequently replaced by less prevalent proteins having a higher binding affinity (Vroman effect [16]). Thus, given that the rate coefficients governing protein binding and unbinding are of suitable magnitude, one may observe an initial, short-lived “soft corona” that subsequently develops into a long-lived “hard corona”. In this context, “long-lived” refers to a residence time of the proteins on the NP surface longer than the duration of the experiment. Depending on the nature of the bonds (covalent, ionic, hydrophobic, etc.) between the protein and NP surface, the characteristic time scales of protein adsorption and desorption can vary widely.

A NP covered by a protein adsorption layer is disguised and, therefore, the initial encounter between the NP and a cell, which may trigger the endocytosis machinery by activating specific receptors [17], is governed by the properties of the protein layer rather than the bare NP surface [2,18–20]. To control the biological effects of NPs (e.g., to accomplish targeted delivery to specific cells or tissues or to inhibit cellular uptake), it is extremely important to understand how the properties of the NP surface can control the structure and dynamics of the protein corona at the molecular level [14,21–34].

In a typical protein adsorption experiment, NPs are incubated with a mixture of proteins, for example, with blood plasma containing thousands of different proteins [35–37]. These proteins all compete for the limited space on the NP surface. The corona may be highly dynamic and vary over time. A precise, quantitative analysis of its protein composition would require measurements in situ to avoid protein adsorption or desorption processes during the experiment. Frequently, however, experimental approaches involve a separation of NPs with adsorbed proteins and proteins free in solution, such as (ultra)centrifugation [36] or size exclusion chromatography [11]. Such approaches will inevitably modify the composition of only loosely adsorbed biomolecules in the corona because these will immediately respond to changes in the local protein concentration by desorption. Thus, only tightly bound proteins, with residence times on the NP surface much longer than the experimental time scale, will be quantified correctly.

**Figure 1:**
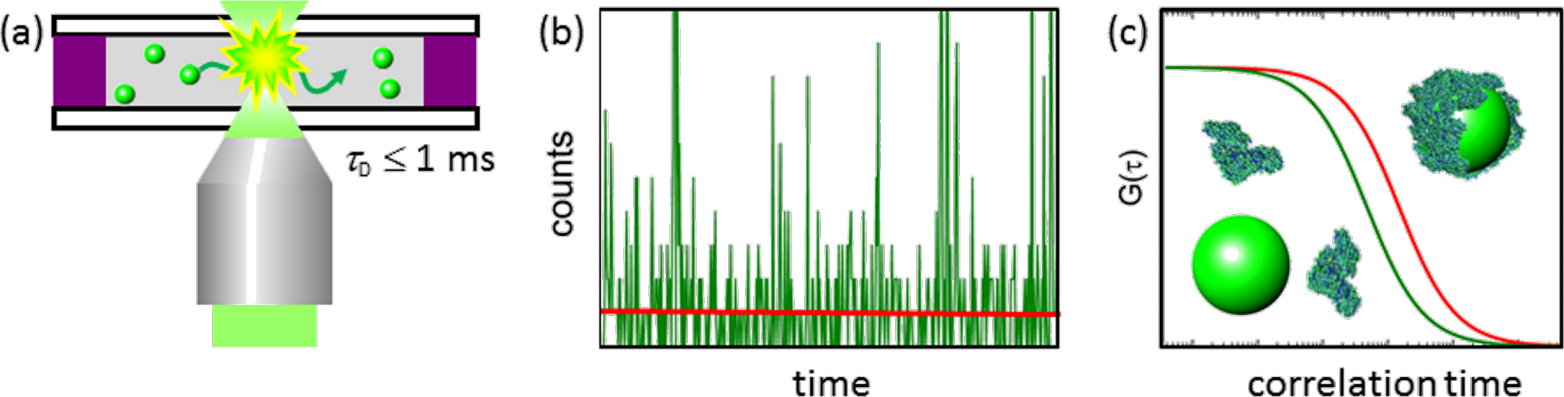
Principle of fluorescence correlation spectroscopy. (a) Individual fluorophores diffusing through the observation volume emit brief bursts of photons. (b) Fluorescence intensity time trace. (c) Fluorescence intensity autocorrelation function. Its amplitude at time 0 scales with the inverse NP concentration; the characteristic decay time, τ_D_, scales with the hydrodynamic radius, *R*_H_, of the diffusing NPs.

We have used fluorescence correlation spectroscopy (FCS) as a powerful method for the in situ study of NP–protein interactions (Figure 1). The technique enables us to measure minute changes in NP diffusivity due to protein adsorption from a solution with a well-defined protein concentration. Consequently, the protein corona formation can be quantified with high accuracy in the presence of free protein [38–39]. FCS experiments were performed on fluorophores dissolved at nanomolar concentrations, which were freely diffusing through the detection volume (~1 fL) of a confocal microscope [40–43]. The fluorescence emission fluctuations were analyzed by autocorrelation to obtain the correlation time, τ_D_, of translational diffusion. We precisely measure the diffusion coefficient, *D*, which changes as protein molecules adsorb onto the NPs, via an increase in τ_D_, by using non-fluorescent proteins and fluorescent NPs. From the Stokes–Einstein equation (see Experimental), the hydrodynamic radius, *R*_H_, of the diffusing particle can be obtained. Sub-nanometer precision is achieved by performing extremely careful measurements and calibration procedures to account for the refractive index mismatch between the sample solution and the immersion medium, viscosity changes of the sample solution with increasing protein content, variations in cover slide thickness and the intensity and shape of the laser beam. Instead of analyzing the translational diffusion of the NPs, FCS experiments can, in principle, also be performed with non-fluorescent NPs and fluorescently labeled proteins to measure concentrations of free and bound protein, as was done by Milani et al. [44]. We have refrained from such an analysis, which requires precise decomposition of the correlation functions into contributions from freely diffusing and NP-bound proteins, because the procedure was not sufficiently reliable in our hands.

In our original FCS study [45], we investigated the adsorption of human serum albumin (HSA) onto carboxyl-functionalized, polymer-encased iron platinum nanoparticles (Fe–Pt NPs) with a hydrodynamic radius, *R*_H_, of 5–6 nm. We prepared HSA solutions of different concentrations in phosphate buffered saline (PBS), pH 7.4, and mixed these solutions, one after the other, with the NP solution (0.1–2 nM) to measure the hydrodynamic radius of the NPs as a function of protein concentration. With increasing HSA concentration, *R*_H_ increased in a single step, indicating that the HSA molecules formed a monolayer around the NPs. The thickness of the monolayer, Δ*R*_H_ ≈ 3 nm, was associated with HSA adsorbing in a specific orientation, namely with one of its large triangular faces. In other FCS studies of adsorption of a single type of serum protein onto NPs (apolipoprotein E4, apolipoprotein A1, apotransferrin) we have also observed monolayer formation without any exception [14,46]. The monolayer thickness could invariably be related to the known molecular dimensions of the proteins, revealing that they adsorbed on the NP surface in a preferential orientation so as to establish Coulomb interactions with the charged NP surfaces [46]. The binding affinities were not governed by the net charge of the proteins (as specified by the isoelectric point) but rather by electrostatic interactions between localized charged regions on the protein and the NP surfaces [46]. An interesting temperature dependence of the binding affinities was observed for HSA and apotransferrin binding to Fe–Pt NPs [30]. In contrast with the naive expectation that a temperature increase should enhance the tendency to dissociate the NP–protein complex into its components (due to an increase of the overall translational entropy), the transition midpoint concentration, *K*′_D_, decreased with temperature, signaling stronger binding. In reality, NP–protein interactions are rather complex and involve contributions from solvent molecules in the proximity of NPs as well. Moreover, the observed behavior could also arise from the larger structural fluctuations of the proteins and/or the polymer shell around the NPs at higher temperatures, which could cause major structural changes forming a free energy-optimized binding interface.

In our earlier work, we used NPs with a chemically well-defined, carboxylic acid-functionalized surface to measure the binding of various proteins. In the present study, we focus on a single protein, again HSA, and explore the change in protein binding onto CdSe/ZnS core–shell QDs with different surface functionalities. These NPs were water-solubilized with small thiolated ligands, leading to thin coatings around the NPs, so that intrusion of proteins into the layer, which may happen with polymeric shells, can be excluded. Cysteamine molecules, which feature primary amine groups, were covalently bound to the QD surface via their thiol groups (see Experimental) to obtain positively charged QDs. Functionalization with dihydrolipoic acid (DHLA) resulted in negatively charged QDs due to the DHLA carboxylic acid group. A surface coating with zwitterionic D-penicillamine (DPA), which has both an amino and a carboxyl functional group exposed to the solvent, created a QD with an overall negative net charge in PBS at pH 7.4 [29]. For comparison, we have also studied commercial QDs with a polymeric carboxylic acid surface functionalization. In the following, we discuss the effects of the different QD surfaces on the HSA binding affinity and the corona thickness and compare these results to those obtained with other NPs.

## Results and Discussion

### QD characterization

Hydrodynamic radii, *R*_H_, of the differently stabilized QDs, determined by FCS and dynamic light scattering (DLS), zeta potentials and fluorescence quantum yields (QY) are compiled in Table 1. The polymer-coated QDs were significantly larger than the other QD preparations because of their thick polymer shells. Experiments with cysteamine-coated QDs were complicated by their strong tendency to adhere to the glass surfaces of the sample holder. We also note that the FCS-determined *R*_H_ values are larger than those measured by DLS. These differences are clearly outside of the error margins for cysteamine- and DPA-coated QDs. QDs are exceptionally bright and show intermittent emission (“blinking”), and one must take great care to avoid optical saturation, which results in an overestimation of the actual size [47–48].

**Table 1:** Characterization of the CdSe/ZnS QDs, suspended in PBS, pH 7.4.

ligand	*R*_H_–FCS (nm)	*R*_H_–DLS (nm)	ζ-potential (mV)	abs./em. (nm)	QY %

cysteamine^a^	6.3 ± 0.4	5.0 ± 0.5	+29 ± 4^b^	592/604	55 ± 2
DPA^a^	6.1 ± 0.1	4.5 ± 0.5	−27 ± 2	592/604	52 ± 2
DHLA	4.8 ± 0.2	4.1 ± 0.7	−31 ± 4	584/604	8 ± 2
polymer^c^	9.3 ± 0.2	8.8 ± 1.7	−27 ± 2	570/586	69 ± 3

^a^Prepared from commercial core/shell QDs (QD ITK 605, Invitrogen); ^b^in milliQ water; ^c^commercial QDs (QD ITK 585, Invitrogen) with carboxyl surface functionalization.

### HSA binding to QDs with different surface ligands

**Figure 2:**
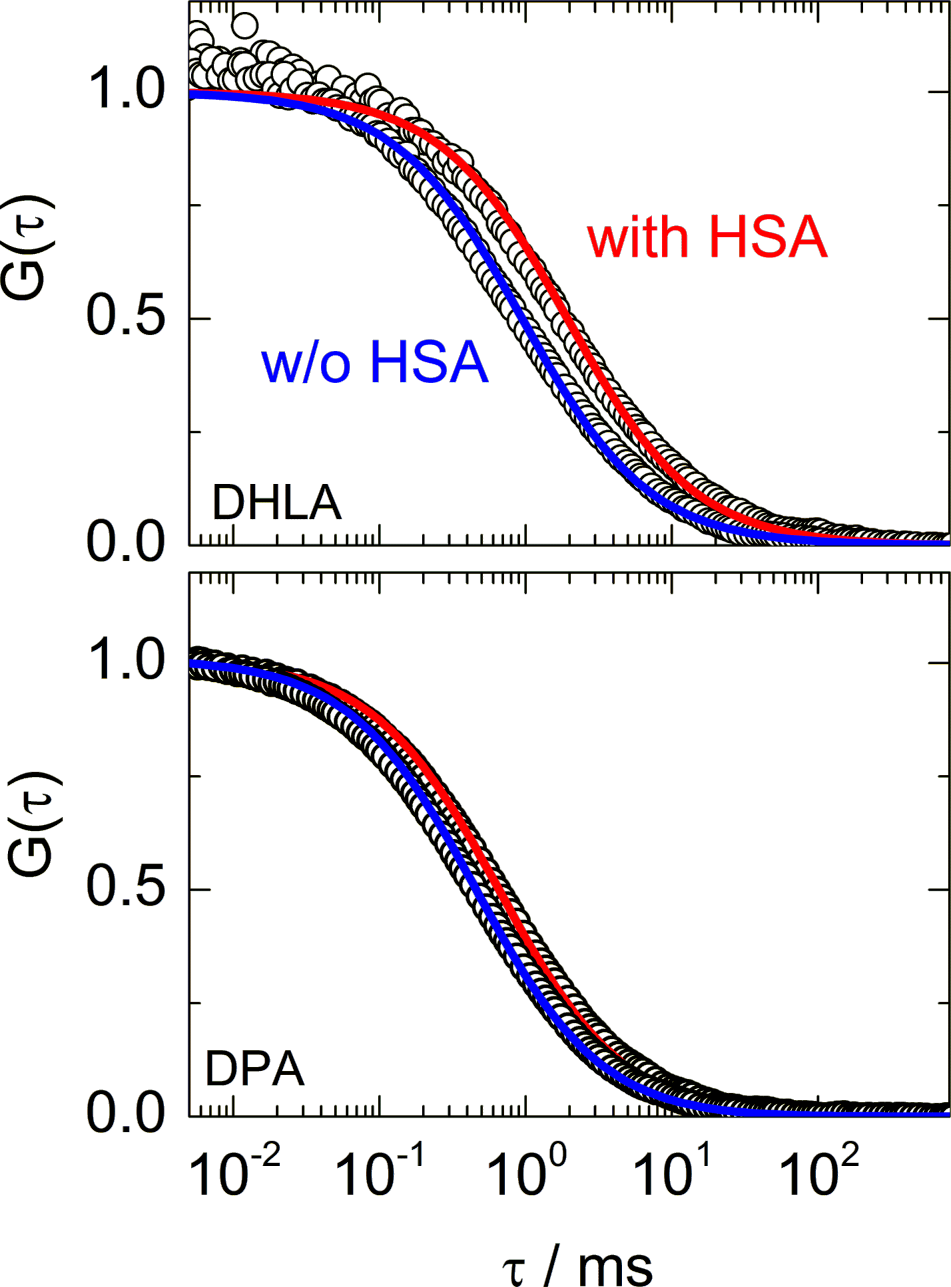
Normalized fluorescence intensity autocorrelation curves of (a) DHLA- and (b) DPA-stabilized QDs dissolved in PBS without (blue) and with (red) HSA at concentrations ensuring complete surface coverage ((a) 800 µM, (b) 40 µM HSA). Symbols: data, lines: fits.

To measure the increase in *R*_H_ as well as the affinity of HSA to the QDs, we recorded FCS autocorrelation curves for QDs freely diffusing in solutions containing HSA at concentrations varying over several orders of magnitude. Nanomolar QD concentrations ensured that only one QD resided in the detection volume on average, so that the intensity fluctuations of NPs within the detection volume, on which the FCS method is based, are large. The HSA concentration was varied on a logarithmic scale in a pre-selected range suitably chosen to observe the transition from uncoated to coated QDs. Exemplary fluorescence intensity autocorrelation curves of DHLA- and DPA-stabilized QDs are depicted in Figure 2. The shifts toward longer times with HSA added to the solution indicate HSA adsorption and the concomitant increase in NP size. The magnitude of the shift is larger for DHLA-coated QDs, implying that HSA binding to these QDs results in a greater radius increase.

**Figure 3:**
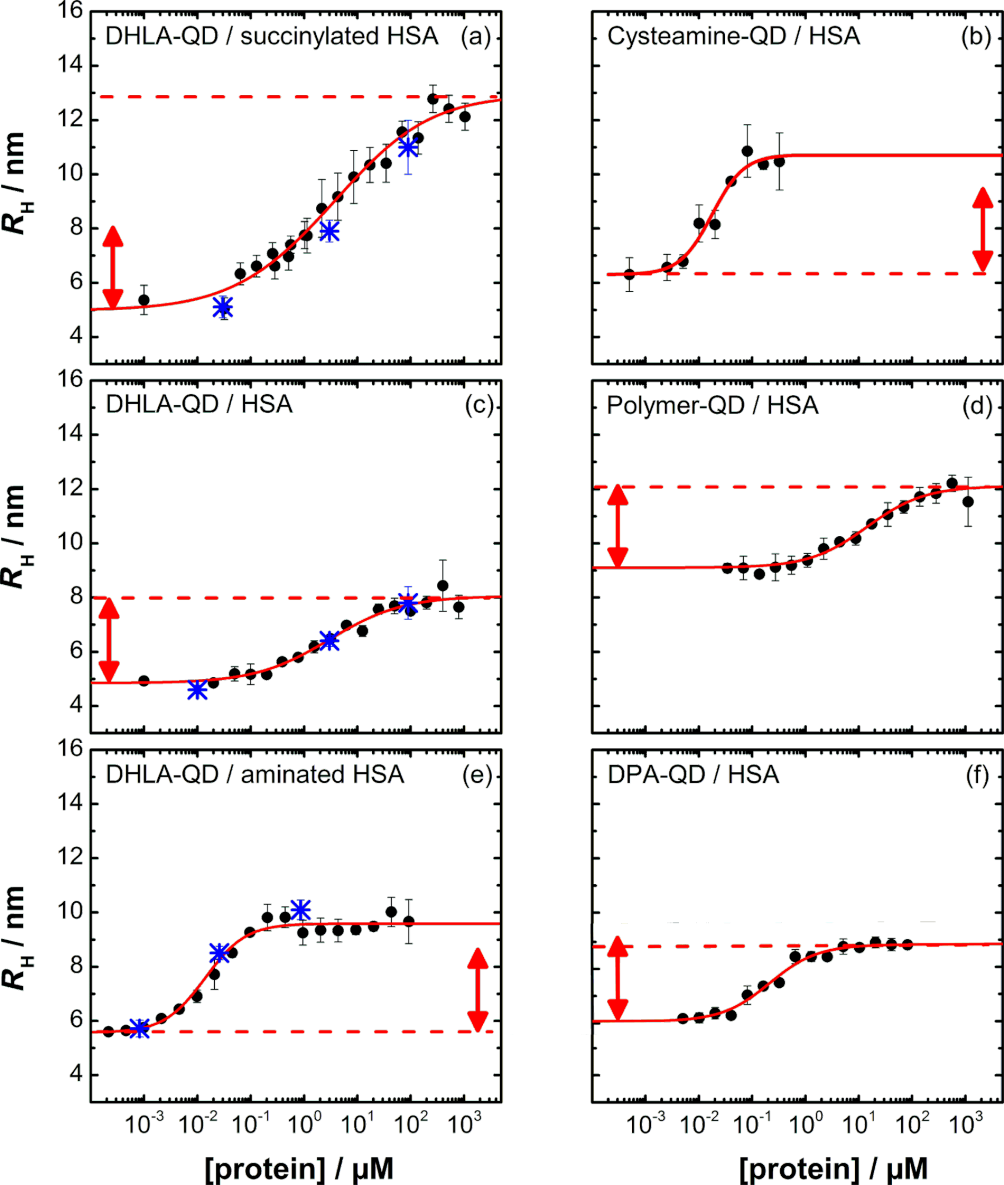
Hydrodynamic radii, *R*_H_, of differently functionalized CdSe/ZnS QDs as a function of the concentration of proteins freely diffusing in solution. Details of the NPs and proteins are given in the individual panels. Data (black circles) were fitted with the model given by Equation 1 and Equation 2 (lines); best-fit parameters are compiled in Table 2. Data in a), c), and e) taken from [22] are included for comparison; blue stars represent *R*_H_ values from controlled desorption experiments. Red arrows with a length corresponding to 3.2 nm are included in each panel to visualize deviations from this value.

Figure 3 shows the increase of *R*_H_ for various CdSe/ZnS QD preparations as a function of the HSA concentration, calculated from the FCS autocorrelation data. Regardless of the QD surface functionalization (and in agreement with our previous studies), *R*_H_ is observed to always increase in a single step. The saturation of *R*_H_ at a higher protein concentration clearly indicates the limited loading capacity of the NPs due to formation of a monolayer of well-defined thickness, Δ*R*_H_. Once the monolayer completely enshrouds the NP, the size remains constant; the tendency to adsorb additional protein molecules is zero. The binding affinity is quantified by the protein concentration at the midpoint of the binding transition, *K*′_D_.

**Table 2:** Parameters describing the adsorption of HSA and chemically modified HSA onto NPs with different surface coatings.

NP/surface charge		*R*_H_ (nm)	Protein	Δ*R*_H_ (nm)	*K*′_D_ (µM)	*n*

Cysteamine–QD	+	6.3 ± 0.4	HSA	4.4 ± 0.4	0.025 ± 0.007	1.5 ± 0.4
DPA–QD	—	6.1 ± 0.1	HSA	2.8 ± 0.2	0.31 ± 0.06	1.0 ± 0.2
QD ITK 585	—	9.3 ± 0.2	HSA	3.2 ± 0.7	24.8 ± 3.8	0.8 ± 0.1
QD ITK 585^a^	—	7.9 ± 0.3	HSA	3.2 ± 0.4	37 ± 12	0.8 ± 0.2
Polymer–Fe–Pt^a^	—	5.6 ± 0.2	HSA	3.3 ± 0.3	5.1 ± 1.3	0.7 ± 0.1
Polymer–Fe–Pt^b^	—	6.0 ± 0.1	HSA	3.3 ± 0.3	9.9 ± 4.7	0.9 ± 0.2
DHLA–QD^c^	—	4.8 ± 0.2	HSA	3.2 ± 0.8	6 ± 3	0.7 ± 0.1
DHLA–QD^c^	—	4.8 ± 0.2	HSAsuc	8.1 ± 0.6	19 ± 8	0.5 ± 0.1
DHLA–QD^c^	—	5.6 ± 0.1	HSAam	4.0 ± 0.1	0.022 ± 0.003	1.2 ± 0.1
DHLA–Au^d^	—	2.9 ± 0.1	HSA	nd	0.08 ± 0.02	0.8 ± 0.1
DHLA–Au^d^	—	4.0 ± 0.4	HSAsuc	nd	1.2 ± 0.4	0.7 ± 0.1
DHLA–Au^d^	—	3.5 ± 0.2	HSAam	nd	0.034 ± 0.008	0.7 ± 0.1
DHLA–Ag^e^	—	1.1 ± 0.2	HSA	nd	0.37 ± 0.02	nd

Data taken from ^a^ [45], ^b^ [46], ^c^ [22], ^d^ [49] and ^e^ [32]. nd = not determined.

The data in Figure 3 were analyzed quantitatively by a simple model presented earlier [45–46]. The NP without or with a partial or complete protein layer is always treated as spherical, so that the dependence of *R*_H_ on the number of bound proteins, *N*, is given by

[1]
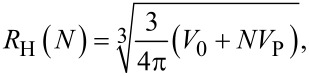


where *V*_0_ is the volume of the NP, and *V*_P_ the molecular volume of an individual adsorbed protein. The dependence of the number of adsorbed proteins, *N*, on the concentration of free protein, [*P*], is modeled by the Hill equation,

[2]
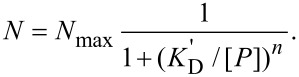


The midpoint of the transition, that is, the concentration of protein free in solution at half coverage in equilibrium, is given by the coefficient *K*′_D_ quantifying the strength of the NP–protein interaction. The Hill coefficient, *n*, contains information about the cooperativeness of binding. In the experiments reported here, *n* is close to but often slightly smaller than one, suggesting that HSA binding is weakly anti-cooperative.

### Orientation of adsorbed HSA molecules

For a variety of prevalent serum proteins, we observed that the thickness of the protein adsorption layer on the NPs correlated with the molecular dimensions of the protein as obtained, for example, from X-ray structure analysis [45–46]. The molecular orientation was shown to be governed by the location of positively charged regions on the protein that preferentially interact with the negatively charged QD surfaces.

**Figure 4:**
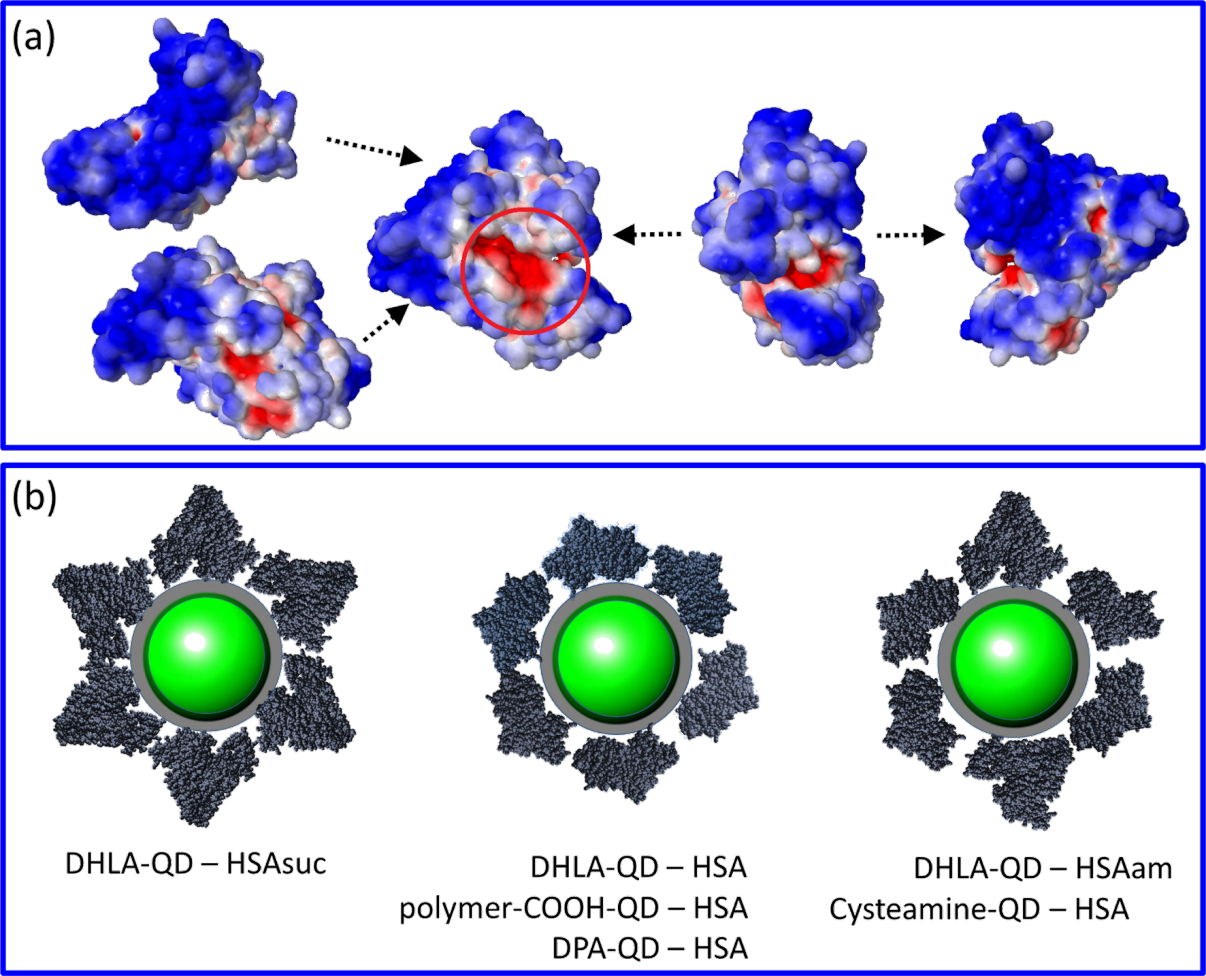
(a) Surface electrostatics of HSA (PDB code: 1UOR) at pH 7.4 (range −5 *k*_B_*T*/*e* to +5 *k*_B_*T*/*e*; calculated online at http://nbcr-222.ucsd.edu/pdb2pqr_1.8 [53]). The potential is represented in graduating colors from dark blue (most negative) to light blue, white, light red to red (most positive). Different views are shown, including the views of the two triangular faces, where the positive region on one of these faces is marked by the red circle, as well as the views of the three smaller sides; arrows indicate the connectivity between the faces. (b) Schematic depiction of HSA monolayers on CdSe/ZnS QDs (green) with different surface coatings. Left: side-on, upright adsorption of a more negatively charged HSAsuc on negatively charged QDs forms a corona of ≈8 nm; center: face-on adsorption via the positive region on negatively charged QDs results in a radius increase of 3.2 nm; right: a corona thicker than 3.2 nm is generated by adsorption in a more random orientation.

The thickness of the HSA corona, Δ*R*_H_, was 3.2–3.3 nm for most NPs (Table 2). HSA is folded into a three-domain structure that can be approximated by an equilateral triangular prism with sides of ≈8 nm and a height of ≈3 nm [50]. Thus, such an HSA monolayer arises if the HSA molecule adheres to the NP surface with one of its two large, triangular faces. We had suggested earlier that HSA adsorption is mediated by electrostatic interactions between a positively charged region on one of the large HSA faces (Figure 4a, red circle) and the negatively charged carboxylic groups on the NP surface [46], as depicted in Figure 4b (center). Table 2, however, also contains a few significantly different Δ*R*_H_ values that deserve discussion. An interesting case is HSA on DPA–QDs, with a radius increase of only 2.8 nm. This value is slightly smaller than expected from the protein structure of HSA, but the excellent data in Figure 3f suggests that we should take this deviation seriously. We cannot envision that HSA binding leads to a layer that is thinner than the physical dimensions of the protein because this would require protein denaturation or replacement of a perhaps incomplete QD coating during protein adsorption. The complete reversibility of the corona formation on DHLA–QDs (see below) suggests that protein denaturation does not occur. A problem with incomplete surface coverage with DHLA is also highly unlikely in view of the negative zeta potential and the excellent colloidal stability of the DHLA-coated NPs. Zwitterionic surfaces, however, are famous for their hydrophobicity, protein adsorption resistance and anti-fouling properties, which strongly argues against a destabilizing effect of DPA-QDs [51]. Of note is that our measured Δ*R*_H_ value is the difference in hydrodynamic radius of NPs with and without protein corona. Consequently, the thickness change can only be attributed solely to the protein if the hydration layer contribution is equal for the bare and protein-bound NP surfaces. DPA–QDs are the only NPs with zwitterionic surface coatings in this data set, and they may have a more tightly bound, thicker hydration layer in the absence of protein. In fact, studies of the hydration layers on proteins, which have similar zwitterionic surfaces, suggest that these layers can extend out to 1 nm from the protein surface [52].

Another interesting case is the adsorption of aminated HSA (HSAam) on DHLA–QDs, for which the overall corona is slightly thicker (Δ*R*_H_ = 4.0 nm, Table 2, Figure 3e) than for the native protein. Random amination of a fraction of the side chains of the acidic residues (aspartate and glutamate) on HSA by ethylenediamine increases the number of positively charged amino functions on the protein surface. The positively charged region on the HSA is maintained upon amination and, in addition, new positive regions will be generated randomly all over the protein surface. Therefore, HSAam will likely adsorb preferentially with its large, triangular faces, but other orientations may also become possible if the additional random charge modifications give rise to another strongly positively charged region. The less uniform HSA attachment to the surface hence results in a somewhat increased corona thickness (Figure 4b, right).

A similar radius increase of 4.4 nm was also found for HSA binding onto positively charged cysteamine-coated QDs (Figure 3b). Here, the protein adsorbs preferentially but also not that regularly with one of its negatively charged triangular faces (Figure 4a) to the positively charged QD surface (Figure 4b, right). We note that the corona thickness is similar to that of positively charged HSAam binding to negatively charged DHLA-QDs, which thus seems to be a similar case with exchanged charge polarities.

Binding of succinylated HSA to DHLA-QDs reveals a significantly thicker protein corona (Δ*R*_H_ = 8.1 nm, Table 2, Figure 3a) [22]. By reacting the amines on lysine side chains with succinic acid anhydride, the number of negatively charged carboxylic acid groups on the surface is increased. Succinylation is likely to reduce the charge density of the positively charged region on HSA that is responsible for binding with the large, triangular face, so that HSAsuc appears to bind preferentially in an upright position (Figure 4b, left).

### NP surface charge and HSA binding affinity

Table 2 also contains affinity data, as quantified by the equilibrium binding coefficient, *K*′_D_, of native and modified HSA binding to QDs with various small ligands, polymer-coated QDs and Fe–Pt NPs as well as DHLA-coated gold and silver nanoclusters (NCs) [49]. Affinity measurements on the latter NPs were performed by measuring their luminescence enhancement due to protein binding rather than by using FCS.

For native HSA, the lowest affinities are found for NPs covered by polymers with carboxylic acid functions (commercial QD ITK 585 and Fe–Pt NPs). This effect is likely due to the polymeric nature of the NP coating giving rise to a larger NP radius and a lower density of carboxylic acid groups (especially for QD ITK 585) in comparison with NPs coated with small thiolated ligands. For the zwitterionic, DPA-coated QDs, *K*′_D_ = 0.31 ± 0.06 µM, the affinity is more than an order of magnitude higher than for DHLA-QDs, presumably because of the additional electrostatic interactions between carboxylic acid side chains on the protein and DPA amino groups on the QD. This is a most interesting finding in the light of the protein adsorption resistance and non-fouling properties that are usually attributed to zwitterionic surfaces [51]. Our data would suggest that these properties may derive from binding a single layer of proteins, which then prevents additional protein aggregation. Clarifying these interesting issues, however, requires further efforts.

The importance of Coulombic interactions is most strikingly revealed upon comparing the affinities of native and charge-modified HSA. Amination and succinylation increases and decreases, respectively, the probability of positively charged regions on the protein surface. Therefore, the binding affinity to negatively charged, DHLA-functionalized NPs (QDs and AuNCs, Table 2) increases in the order HSAsuc < HSA < HSAam. We note that the affinity of HSA toward very small Ag (*R*_H_ ≈ 1.1 nm) and Au (*R*_H_ ≈ 1.6 nm) NCs is much higher than that toward the QDs, which may be due to the different NP sizes. The Au– and Ag–NPs are much smaller than the DHLA–QDs, and even smaller than an HSA molecule. The protein surface appears to be randomly covered with positively and negatively charged regions. The modified proteins, HSAam and HSAsuc, have a larger fraction of positive and negative regions, respectively, than the native protein. A small, negatively charged metal NP is likely to find a small region with a positive surface charge, whereas the larger footprint of a QD will average over negative and positive regions, so that its affinity is lower than that of the small metal NP. If the fraction of positive regions is increased, this selective interaction should become less important. Indeed, we observe comparable, high affinities of the small metal NPs and the larger QDs toward HSAam (Table 2).

A very high affinity, similar to the one of HSAam to negatively charged NPs, was also found for HSA binding to cysteamine-coated QDs, with *K*′_D_ ≈ 0.03 µM. We note that it is very difficult to maintain the condition [HSA] >> [NP] under these high-affinity conditions, which ensures that the concentration of freely diffusing HSA is not altered appreciably upon binding to the NPs. The result of not having HSA in large excess over binding sites is an apparent Hill parameter higher than 1 (here, 1.5 ± 0.4, although it is not that significant in view of its large error), and the real *K*′_D_ will, in fact, be even lower than the one that is quoted.

### Reversibility of corona formation

A quantitative analysis in terms of equilibrium binding isotherms strictly only applies if corona formation is reversible. However, depending on the NP surface, protein adsorption may lead to tight binding, often accompanied by a substantial loss of protein structure. In such cases, desorption can be severely impeded, that is, corona formation is no longer reversible and cannot be treated as an equilibrium process. In fact, most other studies of protein corona formation rely on the separation of NPs with adsorbed proteins from the supernatant and, therefore, are only sensitive to strongly bound proteins, having dissociation rates longer than the experimental time scale [54].

To probe reversibility, we have measured the hydrodynamic radius, *R*_H_, of DHLA–QDs by using FCS. Subsequently, we have incubated the NPs with protein at a concentration that was sufficiently high to ensure formation of a complete corona to determine its thickness, Δ*R*_H_. Afterwards, the protein in the solution was diluted quantitatively up to 10^4^-fold and, after a brief equilibration period, Δ*R*_H_ was measured again. For DHLA-functionalized QDs incubated with HSA, HSAsuc and HSAam, all points of the controlled desorption experiments lay on the respective binding curves within the experimental error, indicating that corona formation was indeed fully reversible (Figure 3a,c,e, blue symbols).

For polymer-coated Fe–Pt NPs, FCS experiments indicated incomplete desorption of HSA, that is, non-equilibrium binding (unpublished data). Considering that the ≈3 nm thick [30] polymer shell consists of dodecylamine crosslinked by poly(isobutylene alt-maleic anhydride) [55], it is highly likely that some HSA molecules get entangled in the polymeric meshwork and may even denature. In general, a protein molecule interacting with a NP may change and even completely denature if the interaction free energy is comparable or larger than the internal energy needed to stabilize its structure. Notably, proteins consist of typically a few thousand atoms but are stabilized by an energy only corresponding to a few hydrogen bonds and, therefore, exhibit large-scale fluctuations at room temperature [56–57]. Thus, already fairly weak interactions can disturb or even unfold proteins. Depending on the details of the NP–protein interaction, a simple equilibrium binding model may in many cases be an oversimplification.

## Conclusion

Because of its relevance for the biomedical field, a huge body of research has been devoted to studies of protein adsorption onto planar surfaces [58–60]. In the light of these findings, it is not surprising that similar processes are observed with NP surfaces. For both planar and NP surfaces, still more knowledge about the detailed physicochemical processes at the molecular level would be highly desirable. In our view, studying protein–surface interactions by using NPs rather than planar surfaces has significant technical advantages, provided that the NPs used in these studies are well characterized. Currently, a thorough physical and chemical surface characterization is still challenging, however, and requires the combination of several methods.

In future studies, the kinetics of protein adsorption, corona stability and aging need more attention. Considering the wide variety of existing NPs and the complexity of biological fluids, substantial variations in their mutual interactions can be expected. Knowledge of these interactions is, however, indispensible for safe applications of NPs in the field of nanomedicine, for example, as highly specific nanodrugs, nanovaccines or imaging tools.

Our studies have provided only a first glimpse at the complexity of NP–protein interactions. A dazzling array of physicochemical parameters modulates these interactions, including NP size, shape, charge, composition, and surface functionalization, protein-specific properties and experimental parameters such as pH, temperature, and solvent composition. To make further progress, it is of utmost importance to perform well-controlled systematic and quantitative studies with well-designed and well-characterized NPs.

## Experimental

### QD synthesis and ligand exchange

**Figure 5:**
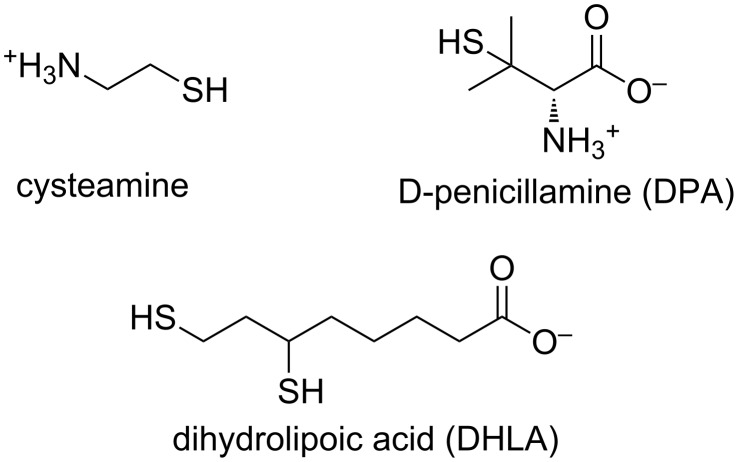
NP surface ligands employed in the present study. They associate with the CdSe/ZnS QD surface via their thiol group(s).

Zwitterionic D-penicillamine (DPA, Figure 5) coated QDs were prepared according to the protocol reported by Breus et al. [61]. Carboxyl-functionalized QDs were obtained by coating CdSe/ZnS QDs with dihydrolipoic acid (DHLA, Figure 5). Briefly, 100 µm DHLA, 20 μL methanol and 0.25 mg CdSe/ZnS QDs were mixed and stirred for 20 min at 60 °C. Subsequently, 500 μL heptane, 300 μL ethanol and 50 μL chloroform were added. The solution was centrifuged for 4 min at 13,500 rpm to precipitate the QDs. The pellet was resuspended in 50 mM tetraborate buffer or 50 mM Tris buffer. Amine functionalization was achieved by mixing 0.5 mg QDs with a deaerated solution of 63 mg cysteamine (Figure 5) hydrochloride in 7 mL methanol. After sonication for 1 h at 60 °C, the solution was cooled to room temperature and centrifuged at 10,000 rpm for 15 min. The pellet was redissolved in milliQ water. If necessary, the centrifugation step was repeated to remove residual organic solvent. Polymer-coated QDs with carboxylic acid surface functionalization (Qdot ITK 585) were purchased from Invitrogen (Carlsbad, CA, USA).

### Particle characterization

#### Optical properties

Absorption spectra were measured on a Cary 100 spectrophotometer (Varian, Palo Alto, CA) and excitation and emission spectra were recorded on a Fluorolog-3 spectro-fluorometer (HORIBA Jobin Yvon, Edison, NJ). Quantum yields were determined relative to rhodamine 6G (R6G) [62].

#### Size and charge determination

Hydrodynamic radii of the ligand-stabilized QDs (dissolved in PBS) and zeta potentials were determined at 20 °C on a Zetasizer Nano-ZS (Malvern, Herrenberg, Germany), using 633 nm light. Note that the 633 nm laser cannot excite the QD fluorescence, as the wavelength is red-shifted with respect to the QD absorption (the first excitation peak of the QDs is centered at ≈580 nm). The results from multiple zeta potential and DLS measurements, that is, the zeta potential and number distributions, were individually fitted with Gaussian functions and then averaged to determine the zeta potentials and hydrodynamic diameters of the particles.

#### HSA modification

**Figure 6:**
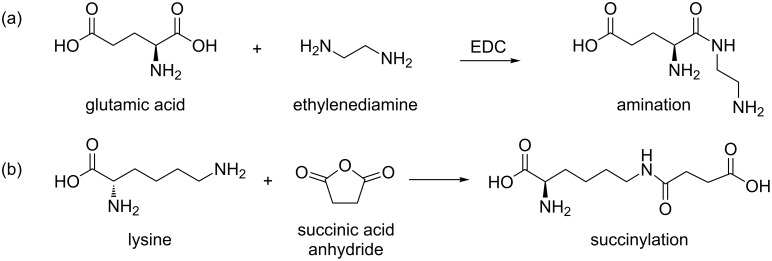
(a) Amination of solvent-exposed glutamic acid and aspartic acid side chains. (b) Succinylation of solvent-exposed lysine side chains.

Succinylation and amination (Figure 6) of HSA (Sigma-Aldrich, Milwaukee, WI) were performed according to published protocols [22,49].

### Fluorescence microscopy

#### Sample preparation

10 min prior to the FCS measurement, 10 µL of the QD solution (≈1 nM) were added to 10 µL of protein solution (at varying concentrations). The mixture was allowed to equilibrate for ≈8 min at room temperature before loading into the sample cell. The cell consists of two standard cover slips (20 × 20 and 32 × 24 mm^2^) separated by two strips of double-sided adhesive tape (thickness 0.2 µm), leaving a 1 mm-wide channel for the sample solution in the middle. For data collection, samples were illuminated continuously for typically 5–10 min; the intensity autocorrelation function was directly computed from the emission intensity.

#### FCS setup

Fluorescence correlation measurements were mainly performed on a custom-built confocal microscope with single-molecule sensitivity [42]. The instrument design is based on an inverted epi-fluorescence microscope (Axiovert 135 TV, Carl Zeiss, Göttingen, Germany). The excitation light (solid-state laser sources, 532 nm or 635 nm, excitation power 6 µW) is routed to the back port of the microscope by a single-mode optical fiber (QSMJ, OZ Optics, Ottawa, Canada). The emitted light is collected by a water immersion objective (UPLAPO 60×/1.2w, Olympus, Hamburg, Germany). It is passed through a dichroic mirror (z532/633xr, AHF, Tübingen, Germany) and suitable band pass filters and, finally, is focused onto a 62.5 µm diameter gradient index fiber (Thorlabs, Newton, NJ, USA) replacing the confocal pinhole. Incoming photons are detected by an avalanche photodiode (SPCM-CD3017, Perkin Elmer, Fremont, CA) and processed by a digital correlator (ALV-5000/E, ALV, Langen, Germany).

Some of the FCS data were taken on a time-resolved microscopy system (Microtime 200, Picoquant, Berlin, Germany) using an inverted epi-fluorescence microscope (IX71, Olympus). Here, the sample is excited by a pulsed, solid state, 530 nm laser (LDH-P-FA-530, Picoquant), sent into the water-immersion objective (UPLSAPO 60XW, Olympus) by a dichroic mirror (z532/635pc, AHF). The fluorescence light is collected by the same objective and focused into a 50 µm pinhole after passing through appropriate filters. For detection, the emission light is split by a 50/50 beam splitter cube and focused onto two avalanche photodiodes (SPCM-AQR-13, Perkin Elmer, Rodgau, Germany). Recorded fluorescence time traces are cross-correlated by commercial software (SymPhoTime, Picoquant). The dual-focus fluorescence correlation spectroscopy setup based on the time-resolved microscope MicroTime 200 was already described elsewhere [39,46].

#### Data analysis

Autocorrelation functions *G*(τ) were analyzed by custom-written software, using a one-species, three-dimensional diffusion model,

[3]
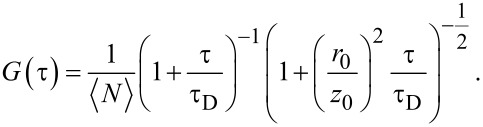



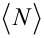
 denotes the average number of particles in the observation volume, which is modeled by a three-dimensional Gaussian function with radial and axial extensions *r*_0_ and *z*_0_.

The diffusion time τ_D_ is related to the translational diffusion coefficient of the NPs, *D* = *r*_0_^2^/4τ_D_. Before fitting the FCS autocorrelation data to the NP-protein association using Equation 3, the FCS curves of the rhodamine 6G reference sample (with known diffusion coefficient, *D* = (4.14 ± 0.05) × 10^−6^ cm^2^ s^−1^ at 25 °C [63]) were fitted (Equation 3, free fit, without any boundary conditions) to determine 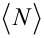, τ_D_ and (*r*_0_/*z*_0_). The average value obtained for (*r*_0_/*z*_0_), 1/8, was kept constant in all subsequent fits of the data.

From the resulting diffusion coefficients, *D*, the hydrodynamic radii were calculated according to the Stokes-Einstein relation, *R*_H_ = *k*_B_*T*/6πη*D*, with solvent viscosity η. Changes in viscosity due to the varying protein concentration were taken into account by using a linear approximation for the contribution of the protein to the solution viscosity, based on an intrinsic viscosity of HSA of 4.2 cm^3^ g^–1^, as specified by the supplier (Sigma-Aldrich).
